# The Armed Forces Health Surveillance Center: enhancing the Military Health System’s public health capabilities

**DOI:** 10.1186/1471-2458-11-S2-S1

**Published:** 2011-03-04

**Authors:** Robert F DeFraites

**Affiliations:** 1 Armed Forces Health Surveillance Center, 503 Robert Grant Avenue, Silver Spring, MD 20910, USA

## Abstract

Since its establishment in February 2008, the Armed Forces Health Surveillance Center (AFHSC) has embarked on a number of initiatives and projects in collaboration with a variety of agencies in the Department of Defense (DoD), other organizations within the federal government, and non-governmental partners. In 2009, the outbreak of pandemic H1N1 influenza attracted the major focus of the center, although notable advances were accomplished in other areas of interest, such as deployment health, mental health and traumatic brain injury surveillance.

## Introduction

The center was established by the Deputy Secretary of Defense in February 2008 [1]. The center’s mission is to promote, maintain and enhance the health of United States (U.S.) military and military-associated populations by providing relevant, timely, actionable and comprehensive health surveillance information. The center is intended to become the central epidemiological resource for DoD. To achieve that aim, AFHSC combined the resources of the Army Medical Surveillance Activity (AMSA), the DoD Global Emerging Infectious Disease Surveillance and Response System (DoD-GEIS), and the Global Health Surveillance Activity supporting the Force Health Protection Directorate in the Office of the Assistant Secretary of Defense for Health Affairs.

This paper outlines the diverse and unique capabilities of the center. As AFHSC matures, evolves and grows, the capabilities and support provided by its legacy agencies are expanding to meet DoD’s needs. The center plays a key role in the collective understanding of infectious disease threats throughout the world, and the impact of these threats on U.S. uniformed and military-associated populations. Today, more than ever, DoD has a more complete picture of the health of American men and women in uniform.

## Background

The center traces its founding to two major policy developments in the 1990s. In the wake of the Persian Gulf War in 1990-91, DoD lacked the ability to address health-related issues in any comprehensive or cohesive manner. Among other initiatives, DoD designated AMSA at the U.S. Army Center for Health Promotion and Preventive Medicine as the department’s Center for Deployment Health Surveillance [2]. AMSA operated and maintained a longitudinal relational database that tracked soldiers’ health-related events throughout their military careers. The effort later evolved into the Defense Medical Surveillance System (DMSS) [3] as an Army Medical Department DoD Executive Agency.

The Defense Department established requirements for documentation of the health status of servicemembers immediately before and following deployment on major operations [4-10]. DMSS was designated as the repository for this information. The Defense Medical Epidemiological Database, a subset of DMSS, is intended for remote user analysis. The database allows authorized users to access de-identified aggregate data on ambulatory visits, hospitalizations and reportable events of active-duty military personnel spanning the previous 10 years. Meanwhile, the DoD Serum Repository contains more than 56 million serum specimens drawn from individuals serving on active duty since the late 1980s [3]. The specimens, maintained at -25 degrees Celsius, are linked with individual DMSS data, as well as augmented by epidemiological studies of military and operational relevance. The information is especially useful for developing precise estimates of prevalence and incidence of infectious diseases in the armed forces.

Another major theme in the years preceding establishment of AFHSC was the recognition of the global health threat posed by emerging and re-emerging infectious diseases [11] and the important role DoD, through its network of clinical, reference and research laboratories in the U.S. and elsewhere, should play in the national effort [12]. To address these needs, DoD-GEIS was established in 1996, with a central hub at the Walter Reed Army Institute of Research. As in the case of AMSA, DoD-GEIS operated as an Army Medical Department DoD Executive Agency.

In 2009, AFHSC and its partner organizations were presented many opportunities to build on this proud legacy and make important health surveillance contributions, many of which are described below in relation to the singular public health event of the year, the influenza H1N1 pandemic.

### Response coordination

Upon recognition of the novel influenza virus H1N1 in April 2009, AFHSC mobilized the military public health community by convening a series of teleconferences linking community of interest, surveillance and public health professionals at armed forces public health centers, Centers for Disease Control, Combatant Commands and staffs of the Service Surgeons General to share information and resources and to standardize the collective approach to surveillance, case reporting and disease control. The center provided a consolidated DoD pandemic H1N1 report on a weekly basis for the remainder of 2009, as the epidemic spread through military population worldwide. Additionally, AFHSC staff augmented those of the Naval Environmental and Preventive Medicine Unit 5 in evaluating an outbreak of pandemic H1N1 aboard the USS Boxer in July 2009.

### Reporting of public health emergencies of international concern

As the Defense Department’s lead public health agency, AFHSC is positioned to play a key role in coordinating the reporting of DoD public health emergencies of international concern (PHEIC), in accordance with International Health Regulations as updated in 2005. In general, DoD is responsible for identifying PHEICs in its population, including events occurring in military communities abroad, and reporting these through appropriate U.S. government channels to the World Health Organization (WHO).

The onset of the influenza pandemic forced the early exercise of this role, and facilitated its clarification as the year continued. DoD Instruction 6200.03 (Subject: “Public Health Emergency Management within the Department of Defense,” March 5, 2010), formalizing this role, had not yet been completed, and the experience of the center through the initial months of the pandemic helped define and resolve some policy issues regarding roles, responsibilities and communication links.

### Development and promulgation of military public health surveillance standards

Public health information becomes more meaningful when data can be compared across multiple agencies over time. Counts, rates and conclusions drawn from the information are rendered more valuable when agencies share common definitions for key terms and adopt standard procedures for calculating important indicators using these definitions.

The center has been charged with leading the U.S. military public health community in the adoption and promulgation of numerous important health surveillance standards. For several years, military medical departments have used a common set of definitions for reportable medical events requiring individual case reports to service public health centers and AFHSC. As a result, this list includes approximately 70 conditions of military public health interest, including infectious diseases, such as malaria and leishmaniasis, as well as noninfectious conditions, such as heat and cold injury and lead exposure. The center coordinated the most recent update of this list, adopted in June 2009 [13], culminating the efforts of a multiservice work group during the previous year. In a similar manner, AFHSC worked in close coordination with staff of the DoD Centers of Excellence for Psychological Health and Traumatic Brain Injury (especially the Defense and Veterans Brain Injury Center), to develop surveillance case definitions for traumatic brain injury.

As the influenza pandemic of 2009 progressed, the surveillance requirements appropriately changed from an initial approach that emphasized individual case finding, laboratory confirmation and documentation of spread to previously unaffected areas, to one that focused on vulnerable populations and clinical severity measures. The center, working with partners in the Services and elsewhere, coordinated DoD’s effort to ensure that the pandemic was tracked in a standard, appropriate and timely manner, while yielding vital statistics required by senior leaders.

### Seminars, exercises and symposia

Epidemiology is AFHSC’s core competency, since another of its mission areas is training and continuing education in epidemiology and public health. The center was especially active during 2009 with symposia on sexually transmitted diseases, H1N1 lessons learned, civil-military cooperation in public health and pandemic influenza tabletop exercises [14]. The tabletop exercises, held in conjunction with the annual Force Health Protection Conference, were targeted to train public health emergency officers and afforded the military medical community with an opportunity to receive continuing medical education credits.

Throughout 2009, AFHSC sponsored rotations for residents in preventive medicine graduate medical education programs at the Walter Reed Army Institute of Research and the General Preventive Medicine residency program at the Uniformed Services University of the Health Sciences. Approximately half of the resident projects resulted in a report published either in the Medical Surveillance Monthly Report or refereed medical literature.

### Epidemiological analysis and reporting

The center provides timely information to a wide spectrum of stakeholders. Its epidemiologists use data in the DMSS—augmented by the Theater Medical Data Store (health outcomes among personnel deployed in southwest Asia), and other datasets as needed—to produce numerous routine and ad-hoc analyses and reports. Routine periodic reports include the DoD Installation Injury Report, featuring specific rates of injuries among the assigned military population on installations (military bases). and DoD Lost Duty Time Application, which similarly includes counts and rates of health events and the resulting cost of these events in terms of lost workdays. Both reports are maintained on the center’s website at http://www.afhsc.mil.

Targeted reports provide statistical support for DoD health quality, deployment health and other important Military Health System metrics. The center also provides analysis products upon request to meet a variety of operational and technical requirements. Finally, AFHSC supports limited research efforts, mission permitting.

A number of reports were developed to support the influenza pandemic, including periodic and ongoing surveillance for adverse events potentially associated with immunization against seasonal or pandemic strains. The Medical Surveillance Monthly Report (MSMR) (http://www.afhsc.mil/msmr), published by AMSA from April 1995 to March 2007, is the center’s most widely read and well-known publication. Its original purpose, as described by AMSA in 1995, is the dissemination of medical surveillance information of broad interest. As stated at the inception of the MSMR, “The ultimate goal … is to provide readily available information necessary to inform, motivate, and empower commanders, their surgeons, and medical staffs to design, implement, and resource programs that enhance health, fitness, and readiness.” [15] That purpose remains relevant today.

Fifteen years and more than 100 issues later, the monthly publication, now published by AFHSC, has emerged as DoD’s public health periodical of record, providing current information and analysis on a wide variety of operational health topics, including trends of communicable and vector-borne diseases, traumatic brain injury, mental and behavioral health issues, and chronic diseases that affect the active-duty military population.

## The future

The center provides DoD with a unique centralized epidemiologic capability to assemble and analyze disease patterns among U.S. military personnel and beneficiaries worldwide. Integral to AFHSC’s role is the ongoing monitoring of the prevalence, incidence and trends of infectious diseases in time, person and place. As a result of these efforts, estimates of operational impact and disease burden can be determined and recommendations can be provided to key decision makers within DoD for implementation of control measures in support of force health protection.

The release of the President’s Policy Directive addressing the Strategy for Countering Biological Threats [16] in November 2009 marked the recognition that strong capabilities in emerging infectious disease surveillance and response provide substantial security value to the nation and the world. With key contacts throughout the world, AFHSC is well positioned to significantly contribute to this greater U.S. government initiative. To further support this initiative, AFHSC will pursue new partnerships to ensure that DoD continues to possess the capability to anticipate, detect and respond to a broad array of threats that could threaten the health of the military and military-associated populations, as well as civilian populations around the world.

## Competing interests

The author declares that he has no competing interests.
